# Percutaneous angioplasty for the management of iatrogenic pulmonary vein stenosis: a case series and brief review

**DOI:** 10.1093/ehjcr/ytaf257

**Published:** 2025-05-26

**Authors:** Saroj Timilsina, Ritesh Chandrasekaran, Michael Saindon, Houman Khalili

**Affiliations:** Department of Cardiovascular Medicine, Memorial Cardiac and Vascular Institute, Memorial Healthcare System, 1150 North 35th Avenue Suite 600, Hollywood, FL 33021, USA; Charles E. Schmidt College of Medicine, Florida Atlantic University, 777 Glades Road BC-71, Boca Raton, FL 33431, USA; Department of Cardiovascular Medicine, Memorial Cardiac and Vascular Institute, Memorial Healthcare System, 1150 North 35th Avenue Suite 600, Hollywood, FL 33021, USA; Department of Cardiovascular Medicine, Memorial Cardiac and Vascular Institute, Memorial Healthcare System, 1150 North 35th Avenue Suite 600, Hollywood, FL 33021, USA

**Keywords:** Atrial fibrillation, Case report, Percutaneous intervention, Pulmonary vein stenosis

## Abstract

**Background:**

Pulmonary vein stenosis (PVS) is a rare complication following pulmonary vein isolation for atrial fibrillation.

**Cases summary:**

We present two cases of iatrogenic PVS, successfully managed with balloon angioplasty and stenting. Both patients experienced significant symptom relief and improved functional status following the intervention.

**Discussion:**

We highlight the importance of early recognition and intervention in PVS, as well as the complexities involved in long-term management, including surveillance strategies and restenosis prevention.

**Learning points**

- Pulmonary vein stenosis (PVS) is a rare but serious complication following pulmonary vein isolation, requiring prompt diagnosis, especially in patients with recurrent procedures.
- Balloon angioplasty with stenting is the standard treatment for symptomatic PVS, offering better outcomes than angioplasty alone.
- Long-term management requires vigilant follow-up to monitor restenosis, with individualized anticoagulation and imaging strategies.

## Introduction

Radiofrequency ablation been established as a safe and durable treatment option for patients with atrial fibrillation (AF). Pulmonary vein stenosis (PVS) is a rare and often under-recognized complication. Here, we describe two cases of PVS successfully managed at our institution with balloon angioplasty and stenting, aiming to shed light on the complexities and nuances of management.

## Summary figure

**Figure:**
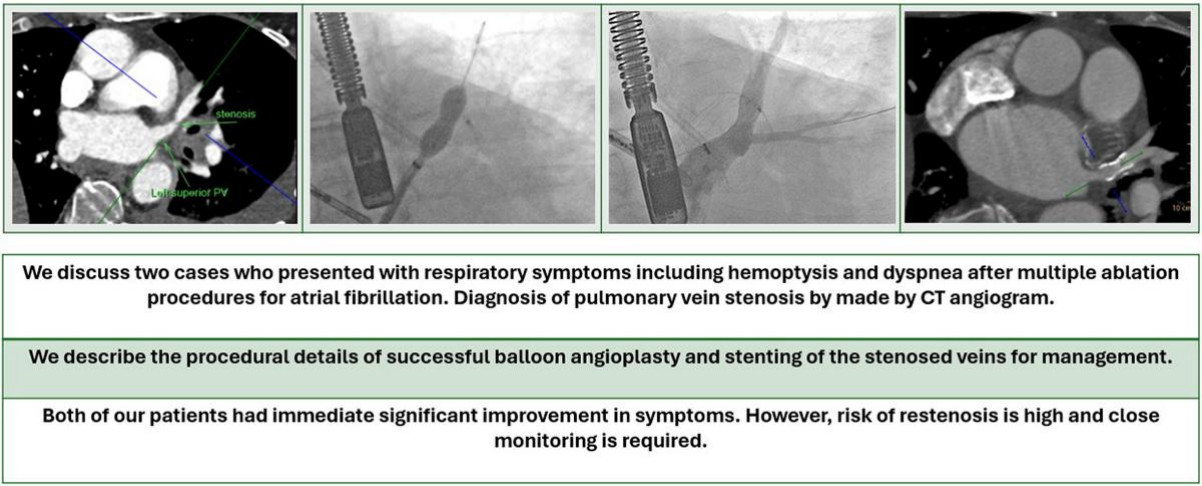


## Case summary

### Case 1

**Figure 1:**
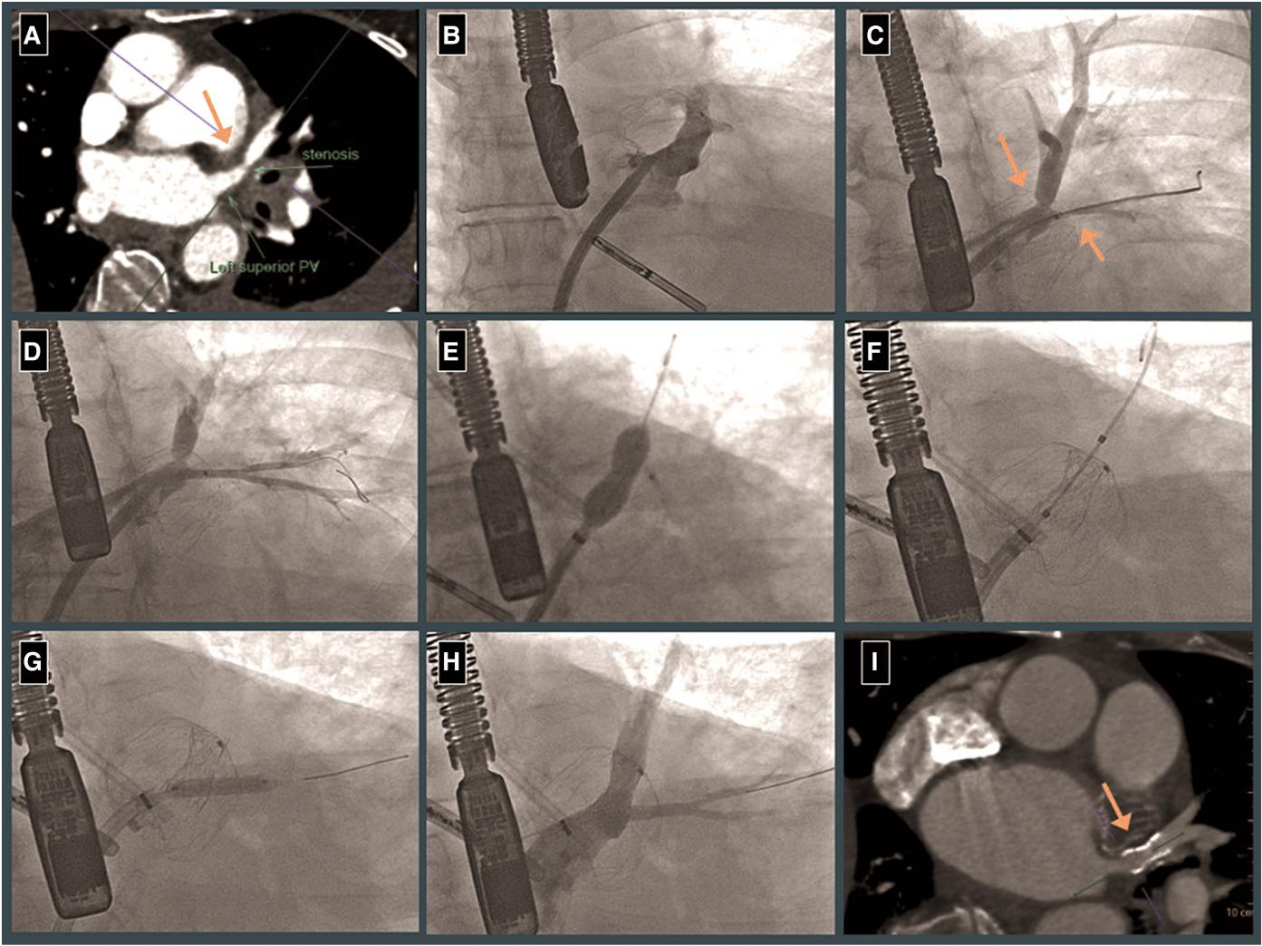
(Left to right). (*A*) CTA showing focal stenosis of the left superior pulmonary vein (arrow). (*B*) Cannulation of the left superior pulmonary vein. (*C*) Pulmonary vein angiogram showing severe stenosis of distal left upper pulmonary vein and ostial lingular vein (arrow). (*D*) Pulmonary vein angiogram after first balloon angioplasty of the lingular vein. (*E*) Balloon angioplasty of the distal left upper pulmonary vein (LUPV). (*F*) Stenting the distal LUPV with 10 × 17 mm stainless steel stent (LAOO device seen in background). (*G*) Repeat balloon angioplasty of the ostial lingular vein. (*H*) Final angiogram showing well-seated stent and patent lingular vein. (*I*) Three month follow-up CT showing patent distal left upper pulmonary vein stent (arrow).

A 56-year-old female with a history of hypertension and symptomatic paroxysmal AF presented with haemoptysis 3 months after her third radiofrequency ablation procedure. Physical exam revealed irregularly irregular pulse but was otherwise normal. Labs were significant for mild anaemia (Haemoglobin: 11.2 gm/dl, normal range: 12–16 gm/dl). A contrast-enhanced computed tomography (CT) of the chest revealed focal stenosis of the left upper pulmonary vein (LUPV), ∼1.5 cm proximal to the ostium (*Figure 1A*), and subsequent bronchoscopy revealed alveolar haemorrhage in the left lingular lobe. Anticoagulation was discontinued, and a left atrial appendage occlusion device (LAOO) was placed. Although she had transient improvement, her haemoptysis recurred, and she was referred for treatment of the left superior vein stenosis.

Under transesophageal echocardiography (TEE) guidance, LUPV was cannulated using a 6 F multipurpose diagnostic catheter and a SafeCross steerable balloon sheath (*Figure 1B*). The Safecross balloon was partially inflated for anchoring and venous angiography. Stenosis of the distal LUPV bifurcation was noted with severe stenosis of the ostial left lingular vein (*Figure 1C*). Left lingular vein was and balloon angioplasty performed using a 4.0 mm balloon (*Figure 1D*). Apicoposterior vein was then stented using a 10 × 17 mm Valeo balloon expandable stainless steel stent (open-cell design) deployed at 6 atm, extending from proximal LUPV into the apicoposterior vein (*Figure 1E* and *F*). Lingular vein was re-wired and stent struts were dilated using a 4.0 × 20 mm Sterling balloon (*Figure 1G*). Final angiography revealed well-seated stent with ‘hour-glass’ appearance, no residual stenosis and brisk flow in all branches (*Figure 1H*). Oral anticoagulation (OAC) was resumed next day of the procedure.

At follow-up visits in 4 weeks and 3 months, she reported complete resolution of haemoptysis despite OAC therapy, and significant improvement of functional status. Surveillance CT Angiogram (CTA) done 3 months post-procedure revealed a patent LUPV stent with no restenosis and possible occlusion of the lingular vein (*Figure 1I*). Given improvement in symptoms we plan to monitor her with periodic screening CTAs guided by symptoms.

### Case 2

**Figure 2:**
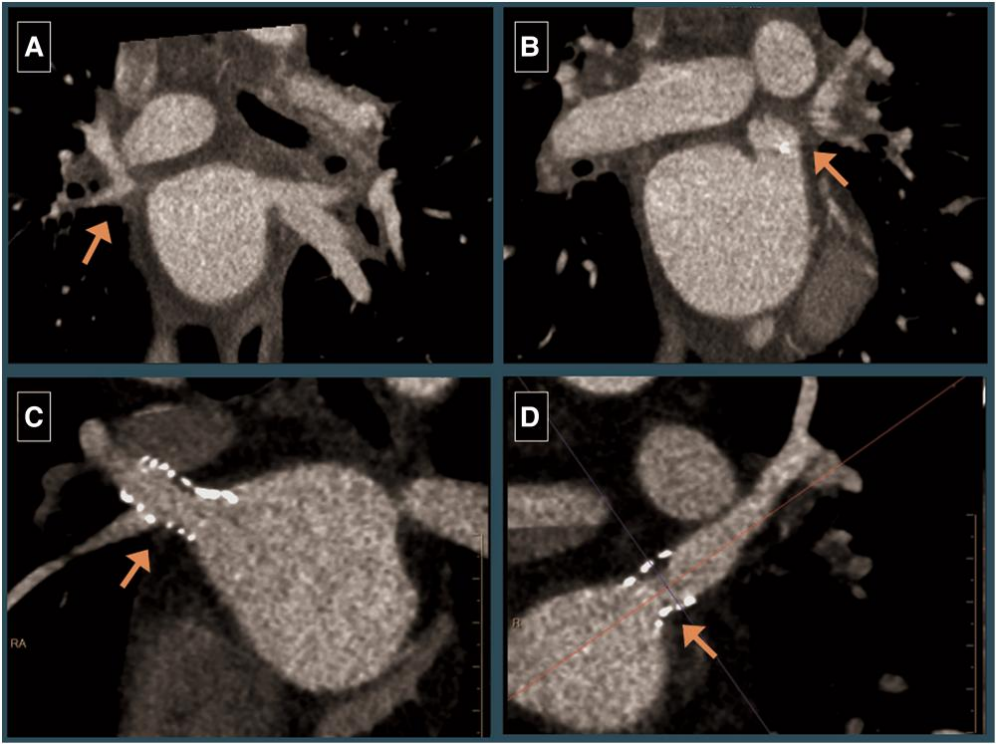
(Left to right). Baseline CTA showing severe stenosis of the right (*A*, arrow) and left (*B*, arrow) superior pulmonary veins. Surveillance CTA 3 months post-procedure showing patient stents in the right (*C*, arrow) and left (*D*, arrow) pulmonary veins.

A 41-year-old male with obesity, sleep apnoea, and paroxysmal AF presented with recurrent symptomatic AF (NYHA III dyspnoea) despite multiple cardioversions and two prior radiofrequency ablations. He also had left atrial appendage closure in the past due to occupational hazard of being on OAC. During his third ablation procedure, severe stenosis of the bilateral upper pulmonary veins (PV) was noted, and the procedure was aborted. A CTA demonstrated occlusion of bilateral upper PVS (*Figure 2A* and *B*), and he was referred for management of PVS. His physical exam was also unremarkable other than irregularly irregular pulse. Complete blood count and comprehensive metabolic panel were within normal range.

**Figure 3:**
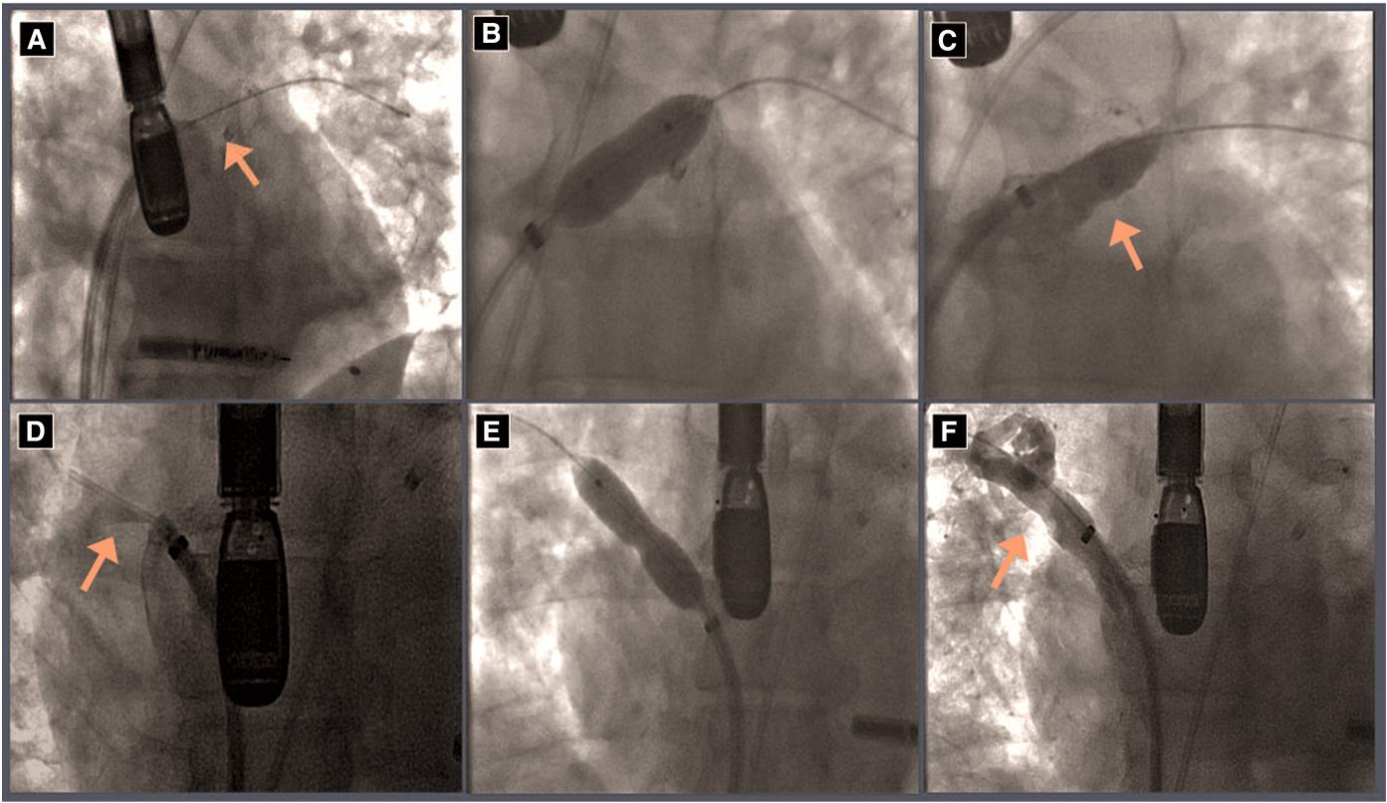
(Left to right). (*A*) Selective angiogram of left upper pulmonary vein showing severe stenosis (arrow). (*B*) Balloon angioplasty of left upper pulmonary vein. (*C*) Final angiogram of left upper pulmonary vein after balloon angioplasty and stenting (arrow). (*D*) Selective angiogram of right upper pulmonary vein (RUPV) showing severe stenosis (arrow). (*E*) Balloon angioplasty of left upper pulmonary vein. (*F*) Final angiogram of RUPV after balloon angioplasty and stenting (arrow).

Angiography demonstrated sub-total occlusion of the right upper PV and total occlusion of the left upper PV. Left upper pulmonary vein was wired and recanalized under TEE guidance, and both veins were stented using 10 mm Genesis XD post-dilated to 11 mm (*Figure 3A–F*). Following the procedure, the patient reported significant improvement dyspnoea and returned to physical activity. He was treated with OAC for 6 months followed by transitioning to dual antiplatelet therapy.

Surveillance CTA was obtained every 6 months and revealed patent stents for the first 12 months (*Figure 2B* and *C*). At the 1-year follow-up, CTA revealed 50%–70% restenosis of the LUPV. Post-dilation of both stents was performed using a 12 mm balloon with excellent angiographic results. The patient remained symptom-free during subsequent visits for 12 months and subsequent CTs demonstrated patent stent with no recurrence of restenosis.

## Discussion

The 2017 Heart Rhythm Society (HRS) expert consensus document on AF ablation defines PVS as a reduction in the diameter of a PV or its branch. It further provides guidance on grading of PVS into mild (<50%), moderate (50%–70%), or severe (>70%) based on the extent of reduction in PV diameter. Despite historical incidence as high as 42%, severe PVS is rare in modern times with studies reporting incidence to be as low as 0.7%. Given its low incidence, guidelines recommend only screening symptomatic patients.^1^

Clinical presentation of PVS varies greatly due to variation in numbers of PVs involved, lesion severity, the response of the pulmonary vasculature to the lesion, time course of stenosis, clinical setting, and the presence and extent of collaterals. Mild to moderate PVS is usually asymptomatic, and symptoms may not be present in all patients with severe PVS. When present, symptoms may range from mild flu like symptoms to dyspnoea at rest and haemoptysis.

Recently, Simard *et al.* suggested a potential workup approach in symptomatic patients with suspected PVS. They recommend obtaining CT/magnetic resonance (MR) PV angiography in symptomatic patients. If CT/MR suggests severe PVS, *trans*-septal PV assessment and/or intervention is recommended. For mild or no PVS, workup for alternative aetiologies is suggested. For patients with intermediate PVS severity, additional investigations including ventilation-perfusion scan, right heart catheterization (RHC)/left heart catheterization (LHC) ± exercise and transthoracic echocardiogram/transesophageal echocardiogram (TEE) are recommended to evaluate if the PVS is severe.^2^ It is important to note that assessing smaller PV stents and subtotal-occluded vessels is challenging on CTA and tends to overestimate degree of stenosis. Invasive assessment with right heart catheterization and selective PV interrogation can help to confirm diagnosis prior to angioplasty. Selective PV angiography can help confirm diagnosis and delineate stenosis severity by visual assessment. Additionally, haemodynamic gradients can also be obtained by passing the catheter beyond the stenosis. Intravscular ultrasonography may be used to provide objective assessment on degree of stenosis before and after intervention.^2,3^

Management of asymptomatic severe PVS remains a grey area. For symptomatic patients, revascularization of the affected PV is the current standard of care. Superiority of balloon angioplasty and stenting over balloon angioplasty alone has been well established in reducing risk of restenosis. In a recent meta-analysis, restenosis rates after stenting was 22.3% compared to 54% with balloon angioplasty alone.^4^ Highest predictor of in-stent restenosis was final diameter of stent <10 mm. Commercially available drug eluting stents for coronary arteries are typically smaller (<6 mm), hence appropriately sized bare metal stents likely provide superior patency. Both of our patients were treated with 10 mm bare metal stent, with the first case inflated to 9 mm (oversized by angiography) and the second case inflated to 11 mm. The stent was further inflated to 12 mm in the second case on follow-up. There are no current guideline recommendations, and this was based on clinical judgment. The advent of drug-coated balloon angioplasty offers an alternative strategy. Few case reports have described successful drug-coated balloon angioplasty of iatrogenic PVS with promising short and long-term results.^5^

Despite excellent immediate post-procedure results, recurrence of symptoms have been described as early as 3.2 ± 2.8 months after index intervention.^6^ The timing and modality of imaging for follow-up is not well described. Although CT venogram can be an excellent tool for larger stents, it is of limited value and often overestimates stenosis in smaller stents. Smaller stents may be better evaluated with invasive PV angiography.^7–9^ We recommend PV CT venogram within first 3–6 months after index follow-up especially if symptoms recur or new symptoms develop. Further testing should be individualized based on prior imaging data and symptoms. The benefit for routine screening in absence of symptoms needs to be weighed against risk of cumulative lifetime radiation exposure and cost benefit especially when data to suggest benefit of treating asymptomatic patients is lacking.

Anticoagulation/antiplatelet strategies following angioplasty or stenting are also not well defined. Prior expert opinion have suggested dual therapy with warfarin and aspirin for at 12 months and individualize lifelong anticoagulation based on stent size/restenosis.^10^ Recently, a small retrospective study suggested lower risk of restenosis in patients on triple therapy (22.7%) compared to those on dual therapy (57.1%).^11^ For our first case, we continued OAC indefinitely and opted not to prescribe additional antiplatelet therapy post-procedure given significant haemoptysis preceding the intervention. For our second case, he was already on an OAC due to need of cardioversions in the recent past. Post-stenting, we added a P2PY12 agent consistent with our practice for patients who undergo coronary interventions. He discussed possibility of stopping OAC around 6 months post-procedure with desire to return to his prior work. Given history of restenosis, we opted to switch him to dual antiplatelet therapy for next 6 months and eventually maintained on single antiplatelet therapy long-term. We recommend carefully weighing in the risk of restenosis to that of major bleeding in each patient and individualize treatment strategies till more data becomes available.

## Conclusion

Severe PVS is a rare but serious complication of pulmonary vein isolation. A high index of suspicion is required for diagnosis, especially in patients with multiple repeat procedures. Dedicated CTA/magnetic resonance angiography aids in early diagnosis. Balloon angioplasty and stenting are the standards of care; however, the risk of restenosis is high. Further studies are warranted to reduce the risk of PVS and identify high-risk individuals.

## Data Availability

All publicly available datasets were fully referenced in the reference list with an accession number or unique identifier such as a digital object identifier (DOI).

## Lead author biography

I am a third year cardiology fellow in Florida, USA. I plan to continue training in cardiac electrophysiology. My prior research has included studying the role of imaging in relation to management of various atrial and ventricular arrhythmias and interventions to prevent complications during ablation procedures.

**Consent:** The authors confirm that written consent for submission and publication of this case report including images and associated text has been obtained the patient, in line with COPE guidance.

**Funding:** None declared.
